# Interactions between Brassinosteroids and Strigolactones in Alleviating Salt Stress in Maize

**DOI:** 10.3390/ijms251910505

**Published:** 2024-09-29

**Authors:** Xinqi Wang, Xue Qi, Zelong Zhuang, Jianwen Bian, Jiawei Li, Jiangtao Chen, Zhiming Li, Yunling Peng

**Affiliations:** 1 College of Agronomy, Gansu Agricultural University, Lanzhou 730070, China; 2 Gansu Provincial Key Laboratory of Aridland Crop Science, Gansu Agricultural University, Lanzhou 730070, China; 3 Gansu Key Laboratory of Crop Improvement & Germplasm Enhancement, Gansu Agricultural University, Lanzhou 730070, China

**Keywords:** maize (*Zea mays* L.), salt stress, hormone interactions, strigolactones, brassinosteroids

## Abstract

Exogenous brassinolide (BR) and strigolactones (SLs) play an important role in alleviating salt stress in maize. We studied the morphological and physiological responses of the salt-sensitive genotype PH4CV and salt-tolerant genotype Zheng58 to BR (1.65 nM), SL (1 µM), and BS (1.65 nM BR + 1 µM SL) under salt stress. Phenotypic analysis showed that salt stress significantly inhibited the growth of maize seedlings and significantly increased the content of Na^+^ in the roots. Exogenous hormones increased oxidase activity and decreased Na^+^ content in the roots and mitigated salt stress. Transcriptome analysis showed that the interaction of BR and SL is involved in photosynthesis–antenna proteins, the TCA cycle, and plant hormone signal transduction pathways. This interaction influences the expression of chlorophyll a/b-binding protein and glucose-6-phosphate isomerase 1 chloroplastic, and aconitase genes are affected. Furthermore, the application of exogenous hormones regulates the expression of genes associated with the signaling pathways of cytokinin (CK), gibberellins (GA), auxin (IAA), brassinosteroid (BR), abscisic acid (ABA), and jasmonic acid (JA). Additionally, exogenous hormones inhibit the expression of the AKT2/3 genes, which are responsible for regulating ion transduction and potassium ion influx. Four candidate genes that may regulate the seedling length of maize were screened out through WGCNA. Respective KOG notes concerned inorganic ion transport and metabolism, signal transduction mechanisms, energy production and conversion, and amino acid transport and metabolism. The findings of this study provide a foundation for the proposition that BR and SL can be employed to regulate salt stress alleviation in maize.

## 1. Introduction

Maize (*Zea mays* L.) is a crucial staple crop globally, with its total production closely linked to living standards and livestock production. Statistics indicate that over 20% of the world’s agricultural land is impacted by land salinization, a figure that continues to rise at an annual rate of 10%. Projections suggest that by 2050, land salinization could affect 50% of the world’s arable land [1,2]. Following soil salinization, plants undergo an accumulation of reactive oxygen species (ROS) [3,4]. Studies have shown that, compared to salt-sensitive genotypes, the salt-tolerant corn genotype exhibits an enhancement in antioxidant enzyme activity in response to salt stress. This enhancement of antioxidant enzymes plays an important role in the removal of ROS in cells [5]. Therefore, to breed salt-tolerant maize varieties, it is essential to understand the physiological and biochemical mechanisms that allow maize to adapt to salt stress. Among these physiological mechanisms, the focus has been placed on those regulated by plant hormones.

To investigate the regulatory effects of exogenous substances on salt stress, various exogenous hormones have been employed. For instance, auxin (IAA), gibberellin (GA), cytokinin (CKs), brassinosteroid (BR), and strigolactones (SLs) are hormones that play active roles in the regulation of growth and development. [6]. Many studies have shown that a single plant hormone does not function independently in plants; rather, it performs complex and multifaceted roles across various developmental stages, tissues, and environmental conditions [7,8].

BR reduces the negative effects of salt stress on plant molecular processes and biochemical mechanisms, thus enhancing the salt tolerance of plants [9]. For example, the overexpression of the BR signaling kinase gene ZmBSK1 (Brassinosteroid SIGNALING KINASE 1) in maize has been demonstrated to enhance tolerance to elevated salt concentrations [10]. In contrast, Arabidopsis mutants lacking the gene responsible for the biosynthesis of BRs exhibited a greater susceptibility to salt stress conditions [11]. However, this phenotype could be rescued by exogenous application of BRs. In terms of interaction, the BRs co-receptor BAK1 (BRI1-associated receptor kinase1) has been observed to interact with the ABA signaling molecule ABI1, ultimately inhibiting stomatal closure [12]. The BZR1 (brassinazole-resistant 1) gene was identified as a regulator of GA biosynthesis and production in apple [13] and carrot [14]. This resulted in the conferral of salt tolerance and petiole cell elongation in plants. The BR signaling pathway does not function in isolation within plants, and how BRs interact with other exogenous hormone pathways to affect plant growth and development and regulate plant response to stress needs further study.

SLs are plant hormones derived from carotenoids and were first discovered as enhancers of parasitic plant seed germination [15]. SLs were found in many stages and parts of plant growth. Studies on rice and pea have shown that SLs inhibit axillary bud growth by affecting OsTB1/PsBRC1 TCP (Teosinte branched1/Cycloidea/Proliferating cell factor) transcription factors, but TCP homologous gene TB1 in maize may act independently of SLs [16,17,18]. In addition, SLs play a role in regulating leaf aging. A positive response to GR24 was found in aging bamboo leaves, and the transcription levels of SL-biosynthesis-related genes MAX1, MAX3, and MAX4 increased in aging leaves, further indicating the important role of SLs in leaf aging [19,20]. While SLs may manipulate a relatively small number of genes compared to other hormones, the potential mechanisms by which SLs interact with other hormones in response to abiotic stress remain to be determined.

Currently, research on the alleviating effects of exogenous SLs and BR on maize salt stress has primarily focused on single applications [21,22]. There are few reports on the combined use of SLs and BR to mitigate salt stress. This study used salt-sensitive genotype PH4CV and salt-tolerant genotype Zheng58 as test materials to investigate the regulatory effects of combined application of SLs and BR on the phenotype and antioxidant properties of maize seedlings under 180 mM NaCl stress. This study aimed to explore the regulatory network of the interaction between SLs and BR under salt stress at the transcriptome level. These results provide important information for understanding the molecular mechanism of salt tolerance in corn and also offer basic data for breeding new varieties with salt tolerance.

## 2. Results

### 2.1. Changes of Maize Leaf Phenotype and Growth Parameters under Exogenous Hormone Treatment

**Figure 1:**
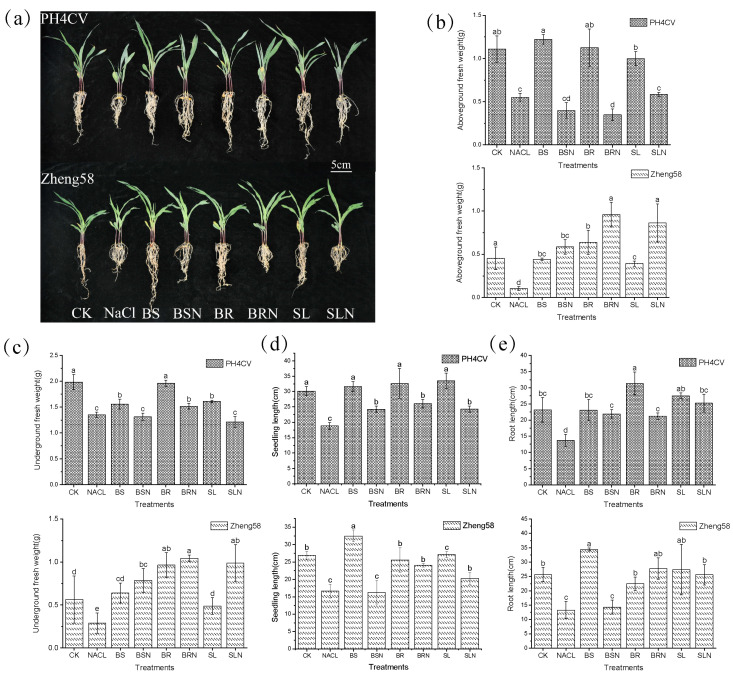
Phenotypes of seedlings under different treatments. (**a**) Effects of exogenous hormones on the morphology of maize seedlings under salt stress. (**b**) Aboveground fresh weight. (**c**) Underground fresh weight. (**d**) Seeding length. (**e**) Root length. Different lowercase letters represent significant differences between treatments by Tukey’s test (*p* < 0.05).

Compared with the control, the growth of the two genotypes with different salt tolerance levels was different under salt stress and exogenous hormone treatment (Figure 1). The salt-sensitive genotype PH4CV and the salt-tolerant genotype Zheng58 both promoted growth to different degrees after the application of hormones, and aboveground fresh weight (AW) and seedling length (SDL) were significantly increased under the treatment of complex hormone BS. Compared with salt stress, underground fresh weight (UW), SDL, and root fresh weight (RW) of the salt-sensitive genotype PH4CV showed significant increase after the application of exogenous hormones under salt stress (*p* < 0.05).

### 2.2. Changes of Antioxidant Enzyme Activities in Maize under Exogenous Hormone Treatment

Compared with the control, salt stress significantly increased superoxide dismutase (SOD), peroxidase (POD), and catalase (CAT) activities in maize leaves. After the application of exogenous hormones, the antioxidant enzyme activity of the two inbred lines also showed an increasing trend compared with salt stress (Table 1).

### 2.3. Changes of Ion Content in Corn Roots and Leaves under Exogenous Hormone Treatment

**Figure 2:**
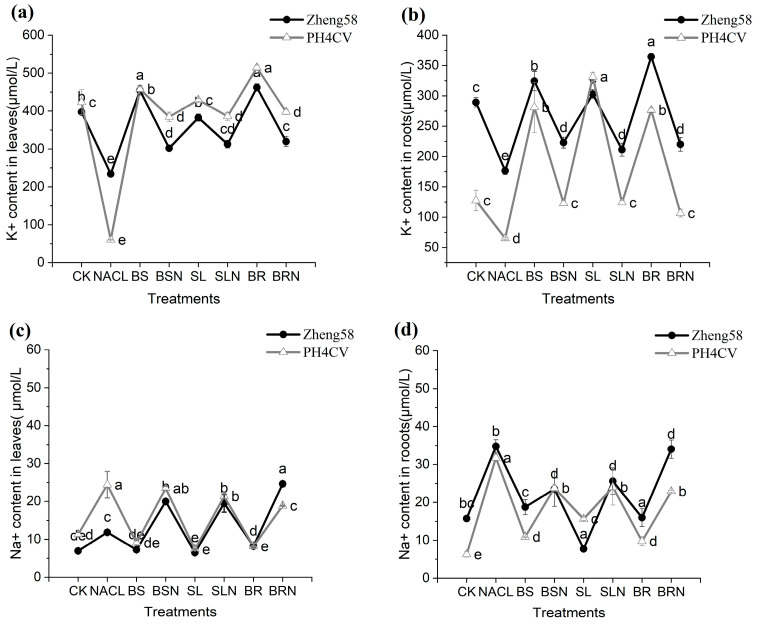
Comparison of Na and K contents in roots and leaves in Zheng58 and PH4CV. (**a**) K^+^ content in leaves. (**b**) K^+^ content in roots. (**c**) Na^+^ content in leaves. (**d**) Na^+^ content in roots. Different letters represent significant within group.

Regardless of salt tolerance, salt treatment resulted in a general increase in Na^+^ and a decrease in K^+^ in roots and leaves (Figure 2). Compared to the control, the Na content in the roots and leaves of both genotypes increased under salt stress. The Na content of the roots of the salt-tolerant genotype Zheng58 was 1.09 times higher than that of the salt-sensitive genotype PH4CV. Following treatment with BSN, SLN, and BRN, the content of Na in the root of Zheng58 was also higher than that of PH4CV, while the difference in Na content between the two genotypes in the leaves was not significant. In the roots, the K content of Zheng58 decreased by 39%, and the K content of PH4CV decreased by 48.9% under salt treatment compared to the control. After the application of exogenous hormones, the K content of Zheng58 in leaves was lower than that of PH4CV. In the roots, only under SL treatment did the K content of Zheng58 fall 11% below that of PH4CV. The salt-tolerant genotype was higher than the sensitive genotype under the treatment of exogenous hormones.

### 2.4. RNA Quality Inspection and Sequencing Results

A total of 48 cDNA libraries were constructed. At the three-leaf (V3) stage, the maize seedling leaves were collected under normal growth, salt stress, and treatments with exogenous hormones SLs and BR, and complex hormone BS under normal growth and salt stress, respectively. A range of 40,915,102–49,196,067 reads was obtained from each sample. After removing the subjoint and low-quality sequencing data, 20,457,551–24,598,033 clean reads were obtained from each sample, with the Q30% value ranging from 93.82 to 95.10%. The GC content range was 52.51–54.80%. The comparison efficiency between the reads of each sample and the reference genome ranged from 81.95% to 91.12% (Table S1). It indicates that the sequencing data across different samples are comparable.

### 2.5. Differential Gene Analysis

**Figure 3:**
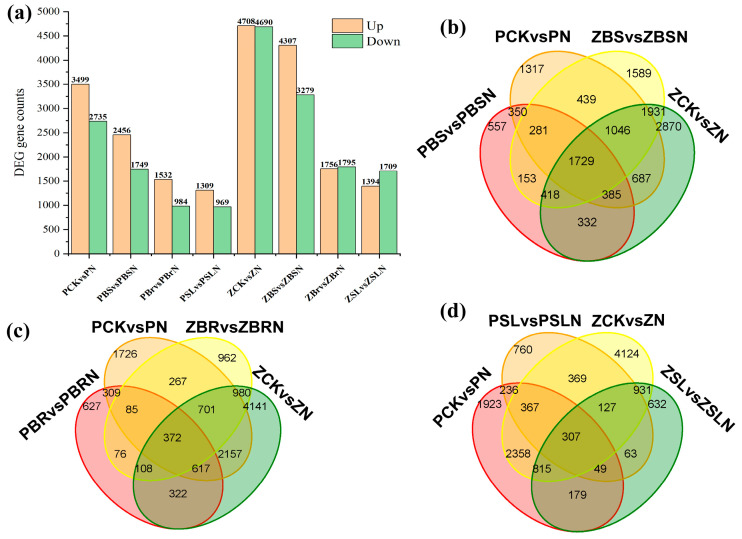
(**a**) The number distribution of up–down DEGs among different comparison groups; (**b**–**d**) Venn diagram analysis of DEGs between different comparison groups.

Under the conditions of fold change ≥ 1.5 and *p*-value < 0.05, DEGs were screened by comparing the gene expression (FPKM) across each treatment combination. The bar chart and Venn chart display the number of up-regulated and down-regulated DEGs in the two inbred lines subjected to salt stress and exogenous hormone remission (Figure 3). Compared with the control, Zheng58 identified 4708 up-regulated DEG expressions and 4690 down-regulated DEG expressions under salt stress, and PH4CV identified 3499 up-regulated DEG expressions and 2735 down-regulated DEG expressions. It was speculated that Zheng58 might enhance its salt tolerance by more up-regulating or down-regulating of DEGs. In the BR vs. BRN and SL vs. SLN comparison groups, 3551 and 3103 differentially expressed genes were identified in Zheng58, and 2516 and 2278 in PH4CV, respectively. In the BS vs. BSN comparison group, 7586 differentially expressed genes were identified in Zheng58, of which 4307 DEGs were up-regulated and 3279 DEGs were down-regulated. In PH4CV, 4205 differentially expressed genes were identified, of which 2456 DEGs were up-regulated and 1749 DEGs were down-regulated.

### 2.6. GO Enrichment Analysis

**Figure 4:**
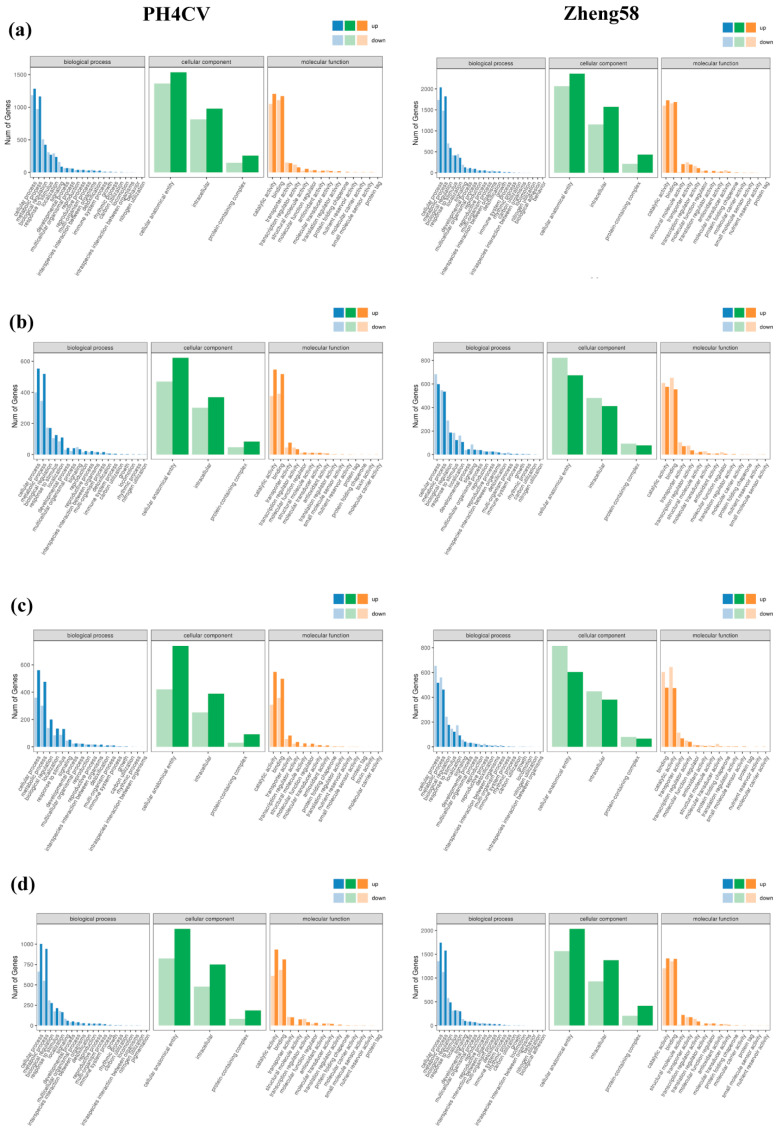
GO analysis of DEGs in two inbred lines under normal treatment and salt stress. (**a**) CK vs. NaCl. (**b**) BR vs. BRN. (**c**) SL vs. SLN. (**d**) BS vs. BSN.

The GO function analysis of differentially expressed genes in two inbred lines under salt stress was divided into three parts: biological process (BP), cellular component (CC), and molecular function (MF). The results showed that in the CK vs. N control group, DEGs were mainly enriched in the cellular process and metabolic process; in BP, the metabolic process was mainly enriched in cellular anatomical entity and intracellular region; and in MF, DEGs were mainly enriched in catalytic activity and binding. Among them, the salt-tolerant genotype Zheng58 has more DEGs (Figure 4a) in these three components than the salt-sensitive genotype PH4CV. Therefore, it is postulated that the variances in the expression of these genes could potentially underlie the disparities in salt tolerance levels between the two inbred lines. In the BR vs. BRN, SL vs. SLN, and BS vs. BSN, the GO functional enrichment terms of the two inbred lines were the same as those of the CK vs. N, but after the exogenous application of BR and SL, the number of up-regulated genes in PH4CV was greater than that in Zheng58 (Figure 4b,c). This indicates that PH4CV is more sensitive to exogenous hormones. In BS vs. BSN (Figure 4d), compared with PH4CV, Zheng58 had a higher number of DEGs in the same enrichment term. It is supposed that BS can mobilize more genes to relieve salt stress.

### 2.7. KEGG Enrichment Analysis

**Figure 5:**
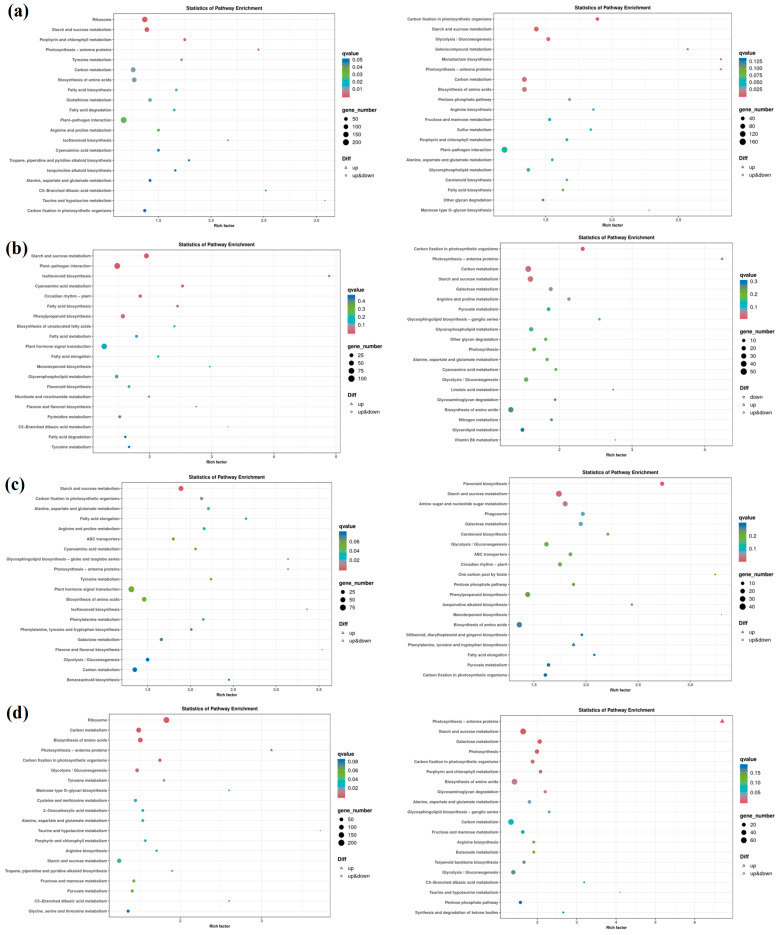
Scatter plot of KEGG enrichment. The horizontal axis represents the ratio of the number of differentially expressed genes annotated to the KEGG pathway to the total number of differentially expressed genes, and the vertical axis represents the KEGG pathway to the total number of differentially expressed genes, representing the KEGG pathway. The size of the points represents the number of genes annotated to the KEGG pathway. The color ranges from red to purple, representing the significance of the enrichment. (**a**) CK vs. NaCl; (**b**) BR vs. BRN; (**c**) SL vs. SLN; (**d**) BS vs. BSN.

KEGG pathway enrichment takes *p* < 0.05 as the threshold of significant enrichment. In this study, the first 20 KEGG pathways (Figure 5) were selected. In the CK vs. N (Figure 5a), the salt-tolerant genotype Zheng58 significantly enriched four pathways, namely, ribosome, starch and sucrose metabolism, porphyrin and chlorophyll metabolism, and photosynthesis–antenna protein. Seven metabolic pathways were significantly enriched in salt-sensitive genotype PH4CV, namely, carbon fixation in photosynthetic organisms, starch and sucrose metabolism, glycolysis/gluconeogenesis, selenium compound metabolism, monopartan biosynthesis, photosynthesis–antenna protein, amino acid biosynthesis, and carbon metabolism. The differences in these metabolic pathways may be the reason for the different genotypes of the two inbred lines.

In BR vs. BRN, the DEGs of Zheng58 were significantly enriched in starch and sucrose metabolism, plant–pathogen interaction, isoflavonoid biosynthesis, cyanoamino acid metabolism, circadian rhythm–plant, fatty acid biosynthesis, and phenylpropanoid biosynthesis. Those of PH4CV were in carbon fixation in photosynthetic organisms, photosynthesis–antenna proteins, carbon metabolism, and starch and sucrose metabolism. The enrichment of the plant hormone signal transduction pathway revealed that the genes responding to exogenous BR were involved in the synthesis and metabolism of IAA, CK, GA, ETH, JA, and ABA. In SL vs. SLN, the only significant enrichment pathway of Zheng58 was starch and sucrose metabolism, PH4CV enrichment of two metabolic pathways, flavonoid biosynthesis, and starch and sucrose metabolism (Tables S4 and S5). The results suggested that exogenous SLs could cope with salt stress via starch and sucrose metabolism.

In the BS vs. BSN control group, Zheng58 significantly enriched six metabolic pathways, namely, ribosome, carbon metabolism, photosynthesis–antenna proteins, carbon fixation in photosynthetic organisms, glycolysis/gluconeogenesis, and amino acid biosynthesis, and PH4CV significantly enriched six metabolic pathways, namely, photosynthesis–antenna proteins, starch and sucrose metabolism, galactose metabolism, photosynthesis, carbon fixation in photosynthetic organisms, and porphyrin and chlorophyll metabolism. It indicated that photosynthesis–antenna proteins and carbon fixation in photosynthetic organisms may be important ways to regulate the interaction between BR and SLs in maize leaves to cope with salt stress (Table S6). By integrating the analysis results of KOG and KEGG, the changes of sodium and potassium ion transmission salt stress signals induced by BS were investigated. It was found that the expressions levels of ion transport genes Zm00001eb288580 (AKT2/3) and Zm00001eb120190 (KAT2), which are involved in the transport of sodium and potassium ions under salt stress, were down-regulated. We believe that the hormone signal and its mutual regulation promote the inward flow of K ions and increase salt tolerance. Additionally, within the pathways of the citrate cycle (TCA cycle) and glycolysis/gluconeogenesis, it was discovered that the glucose-6-phosphate gene and the aconitase gene were down-regulated, and it is hypothesized that they might negatively regulate salt stress in maize.

### 2.8. Weighted Gene Co-Expression Network Analysis (WGCNA) Analysis

#### 2.8.1. Construction of Gene Co-Expression Module

**Figure 6:**
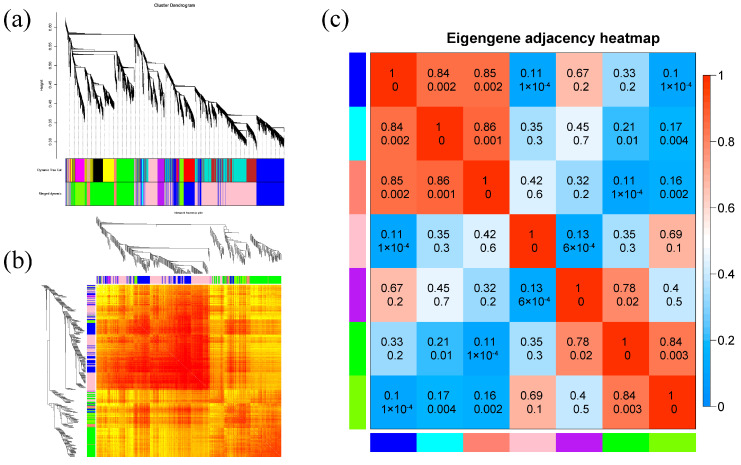
Heat map and cluster analysis of the gene co-expression network. (**a**) Gene co-expression module construction. (**b**) Gene co-expression module heat map. (**c**) Inter-module correlation heat map.

Based on the gene correlation and expression trends of all samples, the gene co-expression network (Figure 6) was constructed using 48 sample genes with FPKM value exceeding 1. A total of seven gene co-expression modules (Figure 6a) were constructed, and the same module had darker color and higher correlation, while different modules had lighter color and lower correlation (Figure 6b), which indicated that the result of cluster analysis was reliable.

#### 2.8.2. Analysis of the Correlation between Modules and Corresponding Phenotypic Characteristics

**Figure 7:**
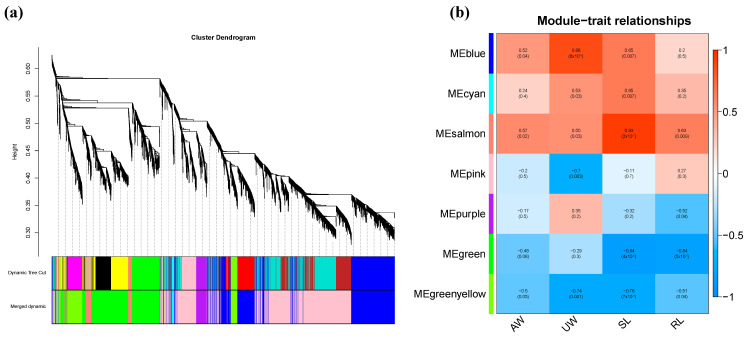
Correlation analysis between modules and phenotypic characteristics. (**a**) Systematic tree map and correlation heat map of genes. (**b**) Heat map of module and character correlation.

In this study, the traits measured were subjected to association analysis with differential genes to identify differential gene modules significantly associated with these traits. The traits of AW, UW, SDL, and RL in maize seedlings were found to have significant positive correlations with the blue module and salmon-colored module, and the correlation coefficients were 0.57, 0.88, 0.93, and 0.63, respectively (Figure 7).

#### 2.8.3. Functional Analysis of Gene Module

**Figure 8:**
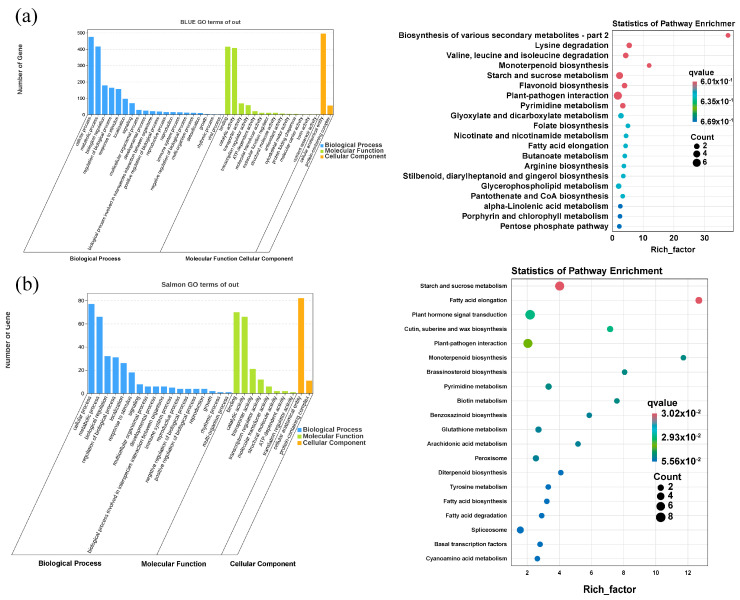
GO and KEGG annotation analysis of two related gene modules. (**a**) Blue module gene GO and KEGG annotation analysis. (**b**) Salmon-colored module gene GO and KEGG annotation analysis.

GO and KEGG analyses of genes within the blue module and salmon-colored modules with high correlation coefficient are shown in Figure 8. The biological processes were the metabolic process, single organism process, cellular process, and biological regulation. The cellular components were fine cells, organelles, cellular parts, and membrane components, and molecular functions were binding, catalytic activity, transport activity, and nucleic acid binding transcriptional factor activity. KEGG annotation showed that the genes in the blue module were mainly enriched in the biosynthesis of various secondary metabolites—part 2, lysine degradation, and valine, and the genes in leucine and isoleucine degradation were monoterpenoid biosynthesis, starch and sucrose metabolism, lavonoid biosynthesis, plant–pathogen interaction, and pyrimidine metabolism; the salmon-colored module was significantly enriched in starch and sucrose metabolism and fatty acid elongation.

#### 2.8.4. Visualization Analysis of Core Genes of the Gene Module

**Figure 9:**
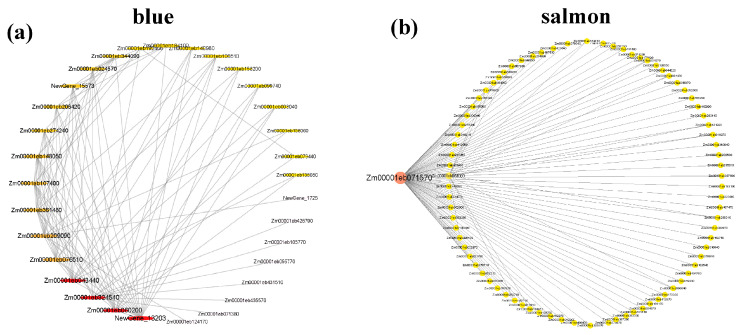
Interaction analysis of the core gene network in the module. (**a**) Blue module; (**b**) salmon-colored module.

In order to mine Hub genes related to traits, cytoscape software_v3.10.0 [23] was used to visualize the co-expression network of a single co-expression module. In this study, the top 100 genes (including duplicates) weighted by blue- and salmon-colored modules were visualized and analyzed by gene connectivity analysis (Figure 9). The core genes of the blue module mainly included Zm00001eb060200, Zm00001eb324540, and Zm00001eb043440. The main core gene of the salmon-colored module was Zm00001eb408430. The gene annotations were as follows: probable glutamate carboxypeptidase VP8, protein phosphatase 2C 1, cytochrome b5, and amino acid permease 3. Respective KOG notes concern inorganic ion transport and metabolism, signal transduction mechanisms, energy production, and conversion, as well as amino acid transport and metabolism (Table S2).

### 2.9. RT-qPCR Verification

**Figure 10:**
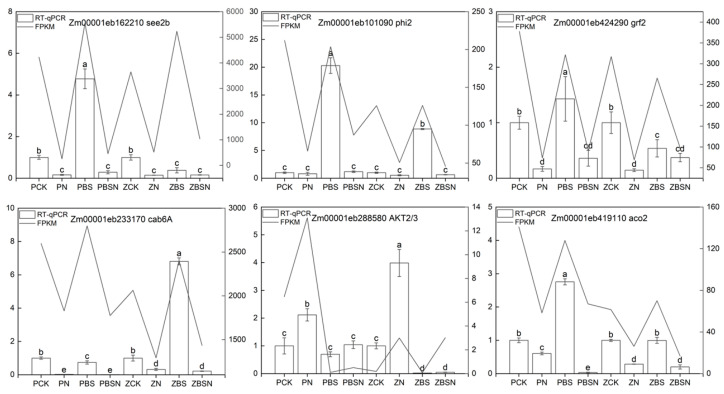
Validation of eight randomly selected genes by quantitative real-time PCR (RT-qPCR). The mRNA expression levels were normalized to the expression level of ACTIN, and the means from three biological replicates are shown. Bar charts and line charts represent RT-qPCR and RNA-Seq data, respectively. Different letters represent significant within group.

To confirm the RNA-Seq results and investigate the candidate genes that play a role in the salt tolerance pathway regulated by BR and SLs, we chose four differentially expressed genes (cab6A, aco2, phi2, and AKT2/3) for validation in the BS vs. BSN comparison. We randomly selected two other DEGs (grf2 and see2b) for validation using quantitative reverse transcription polymerase chain reaction (RT-qPCR) (Figure 10). The RNA sequencing results were consistent with the RT-qPCR results, indicating the reliability of the sequencing results.

## 3. Discussion

### 3.1. Physiological Response of Exogenous Hormones to Maize under Salt Stress

Salt-concentration-induced osmotic and ionic stress leads to oxidative damage, which leads to the accumulation of reactive oxygen species. This accumulation adversely affects photosynthesis and cell membrane integrity, thereby affecting plant growth and development, ultimately resulting in reduced yield [24]. The results demonstrated that the growth of both inbred lines was inhibited under salt treatment. Following the application of SLs and BR alone, the indicators of Zheng58 and PH4CV exhibited an overall increasing trend compared to the salt stress, with the increasing trend of the PH4CV indicators being more pronounced under hormone treatment. Additionally, after the application of BS, the traits of both inbred lines showed a significant upward trend relative to salt stress, with the salt-tolerant material Zheng58 displaying growth traits similar to those of the control group under BSN treatment. The recovery of growth phenotype proved that the compound hormone could alleviate salt stress, and it was speculated that there might be a synergistic effect between SLs and BR. The first line of defense of plants against reactive oxygen species is that SOD will convert superoxide anions into H_2_O_2_, and POD and CAT can convert H_2_O_2_ into H_2_O and O_2_ [25]. The activities of antioxidant enzymes in maize seedling leaves were quantified in this study. There was a tendency for the activities of SOD, POD, and CAT in maize leaves to increase after the topical application of SLs, BR, and BS solutions compared with salt treatments (180 mM). This suggests that appropriate concentrations of exogenous SLs, BR, and BS can modulate the activities of antioxidant enzymes and help maize seedlings cope with oxidative stress under stress. This is consistent with findings in rice [26] and sunflower [27].

Plants use different strategies to deal with excessive Na^+^—one is to isolate Na^+^ into vacuoles, and the other is to recover Na^+^ from transpiration flow through specific transporters to prevent Na^+^ transfer to young leaves [28,29]. The results showed that under salt stress, the content of K^+^ in maize leaves increased significantly, and the content of Na^+^ decreased significantly; especially under SL treatment, the content of Na in roots of Zheng58 decreased significantly, which was the same as that of SLs in soybean seedlings [30]. It is speculated that SL is beneficial to promote Na transport in roots, which helps maize maintain ion balance under salt stress and reduce the effect and development of salt stress on maize growth. In rice, it has been observed that the ability of Na^+^ exclusion in varieties with varying salt tolerance is dependent on the external concentration of K^+^ [31]. This also explains why Zheng58 exhibited a lower K content in its leaves compared to PH4CV under hormone treatment, as Zheng58 was unable to maintain a high Na/K ratio when faced with K^+^ depletion. To conclude, we hypothesize that there might be a synergistic effect between SLs and BR.

### 3.2. Analysis of Tolerance of Two Inbred Lines to Salt Stress

#### 3.2.1. Effect of BR on Differential Gene Expression in Maize under Salt Stress

Plant hormone signal transduction plays an important role in plant defense mechanisms against a variety of biotic and abiotic stresses. BR regulates salt tolerance in plants by interacting with other plant hormones [32]. The transcriptome analysis showed that in the two inbred lines sharing DEGs, the application of BR suppressed the expression of the AUX/IAA gene within the auxin signaling pathway, the B-ARR (type-B ARABIDOPSIS RESPONSE REGULATORS) gene in the cytokinin signaling pathway, and the expression of COI1 (CORONATINE INSENSITIVE1) and JAZ (Jasmonate ZIM-domain) in the JA signaling pathway. The expressions of the GA signaling pathway GID1 (GIBBERELLIN INSENSITIVE DWARF1) receptor gene and ETH signaling pathway CTR1 (CONSTITUTIVE TRIPLE RESPONSE) gene were up-regulated. Conversely, the receptor PYR/PYL gene in the ABA signaling pathway was inhibited, while the expression of SnRK2 (sucrose nonfermenting 1-related protein kinases) gene in this pathway was promoted. In addition, in the BR signaling pathway, the expression of the TCH4 (xyloglucan endotransglucosylase by sequence similarity and enzyme activity) gene was down-regulated in Zheng58 but up-regulated in PH4CV. The BRI1 (brassinazole resistant 1) gene was repressed.

It has been demonstrated that ABA inhibits BR signaling under salt stress, suggesting that BR antagonizes ABA [33]. This study demonstrated that the inhibition of ABA synthesis by BR resulted in an increase in SnRK2, which maintained osmotic homeostasis by retaining water and promoting stomatal closure. GID1 activates the degradation of the DELLA (asp-glu-leu-leu-ala) protein [34], while JA increases the stability of the DELLA protein through the degradation of JAZ, thereby promoting stem elongation. B-ARRs (type B response regulators) have been reported to be under post-transcriptional control at the transcriptional level [35]. This process also involves cross-regulation of multiple hormonal signals: targeting genes encoding key transcription factors such as PIFs, BES/BZR, and ERFs, genes for BRI1/BAK1/BAK743, PYR/PYLs44, and TIR1/AFB2 phytohormone receptors, and negatively regulating AUX/IAA in growth hormone signaling, EBF1/2 in ETH signaling, BIN2 in BR signaling, and DELLA proteins in gibberellin signaling [36]. CTR1, a negative regulator of ETH signaling, has been demonstrated to play a positive role in increasing salt tolerance in Arabidopsis [37]. Furthermore, it has been shown that ETH and BR act in a synergistic manner in alleviating salt stress. BRI1 promotes BR-mediated ETH production, and exogenous application of BR has been shown to induce the production of H_2_O_2_ and ETH accumulation. Furthermore, the two molecules appear to positively regulate each other, resulting in enhanced antioxidant enzyme activities under salt stress [38]. However, an excess of ETH can impede plant growth [39]. The crosstalk among hormones in the BR signaling pathway in this experiment may have resulted in the production of ETH content, which could potentially promote the alleviation of salt stress in maize. TCH4 has been demonstrated to regulate cell wall homeostasis [40]. We believe that the differential expression of TCH4 in Zheng58 and PH4CV is attributable to variations in sensitivity to exogenous BR between the two inbred lines.

#### 3.2.2. Effect of SLs on Differential Gene Expression in Maize under Salt Stress

In this study, physiological analysis showed that externally applied SLs could positively regulate salt tolerance in maize. Transcriptome analysis revealed that the two inbred lines shared DEGs with up-regulated expression of AUX/IAA, TCH4, and PP2C, alongside down-regulated expression of PYR/PYL and MYC2.

The Aux/IAA gene family is a transcription factor in plants, one that plays a regulatory role in plant growth by modulating auxin levels. Some studies have shown that IAA24 is related to the synthesis of the secondary cell wall [41]. In this study, Zm00001eb301590 (IAA24-auxin-responsive Aux/IAA family member) was up-regulated in two inbred lines. Additionally, it was found that bZIP transcription factor (Zm00001eb176680) was up-regulated. The bZIP transcription factor bZIP11 plays a role in maintaining energy homeostasis and regulating primary root growth by directly activating IAA3 transcriptional reprogramming. IAA3 is an inhibitor of root growth [42]. In the phenotypic map, it was also obvious that the number of lateral roots under SL treatment was less than that under BR treatment (Figure 1a). Anthocyanin regulatory gene MYC2 functions in conjunction with IAA to regulate lateral root growth [43]. In Caragana korshinskii, CkMYC2 was found to act directly with CkPYL4 and CkPP2C in leaves [44], resulting in a reduction in JA levels. In this study, down-regulated expression of MYC2 (Zm00001eb429330) may inhibit JA accumulation and promote maize growth. SL down-regulated the PYR/PYL receptor gene Zm00001eb013780 (abscisic acid receptor PYL4-like) and activated the downstream target of PP2C, which may lead to stomatal closure.

#### 3.2.3. Effect of BS on Differential Gene Expression in Maize under Salt Stress

The interaction between hormones plays a complex and efficient role in coping with abiotic stress.

Chlorophyll is essential for photosynthesis and plays a vital role in the growth and development of plants. Studies have demonstrated that oxidative stress can disrupt photosynthesis and upset the electron transport chain in chloroplasts, thereby increasing the production of ROS, causing oxidative damage, and accelerating plant senescence [45]. Chlorophyll a/b-binding protein (CAB) is the apolipoprotein of the photosystem II (PSII) light collection complex in higher plants [46]. CAB is normally part of the antenna complex [47]. Silva et al. found that the expression of chlorophyll a/b-binding protein is affected by abiotic stress, which indicates that the CAB gene plays an important role in abiotic resistance [48]. In previous studies, it was found that the up-regulation of chlorophyll a/b-binding protein expression enhanced the accumulation and stability of chlorophyll in the chloroplast thylakoid membrane of green tomato fruit [49]. In this study, we found that BS up-regulated the expression of genes related to CAB (Zm00001eb168100, Zm00001eb233170, Zm00001eb325410, Zm00001eb324240, Zm00001eb320890, Zm00001eb357740, Zm00001eb112260, Zm00001eb433540, Zm00001eb107560, Zm00001eb100140, and Zm00001eb324090). The expression of CAB in senescent kiwifruit leaves was down-regulated. However, melatonin treatment increased the expression of chlorophyll a/b-binding protein and delayed the senescence of kiwifruit leaves [50]. It is speculated that exogenous BS can slow down the degradation of chlorophyll, which is beneficial to the accumulation of soluble protein and soluble sugar, so as to maintain the balance of cell metabolism. RT-qPCR was used to verify the expression of Zm00001eb233170 (chlorophyll a/b-binding protein 6A, Cab6A) (Figure 10).

Transcriptome analysis demonstrated the influences of BS on hormone signaling pathways in the two inbred lines: GH3 and ARF genes in the IAA signaling pathway were down-regulated. The expression of PYR/PYL genes was down-regulated in the ABA signaling pathway, whereas PP2C, SnRK2, and ABF genes were up-regulated. GID1 was up-regulated and the DELLA protein was down-regulated in the GA signaling pathway. CTR1 and EBF1/2 were up-regulated and the EIN3 genes were down-regulated in the ETH pathway. BAK1, BRI1, and BZR1/2 genes were down-regulated in the BR signaling pathway, and the TCH4 gene was up-regulated. The JAR1 (jasmonic acid resistant 1) gene was down-regulated in the JA signaling pathway. JAR belongs to the GH3 class I protein, catalyzing the combination of JA with isoleucine (Ile) to produce jasmonic acid–isoleucine (JA-Ile). JA-Ile facilitates the degradation of JAZ by the 26S proteasome, thereby activating transcription factors such as MYC and MYB, directly regulating JA response genes and thus influencing cell growth [51]. PIF4 (phytochrome-interacting factor 4) was initially discovered as a negative regulator of the phytochrome signal pathway. Photosensitive pigments detect red and far-red light and promote light-induced changes in plant growth through complex regulatory mechanisms [52]. BR integrates various environmental factors, such as light and temperature, to regulate cell growth. BZR1 directly interacts with PIF4 and controls a subset of downstream gene expression that overlaps with PIF4’s targets [53]. Under light conditions, in response to both steroid hormones and environmental cues, the interaction between BZR1 and PIF4 regulates a fundamental transcriptional network that controls plant growth [53]. Simultaneously, the expression of the leucine-rich repeat F-box protein MAX2/D3/RMS4, one of the SL receptor proteins, is up-regulated. This suggests an interaction between the MAX2 receptor and Aux/IAA protein, leading to the degradation of Aux/IAA protein through the protein degradation pathway. This, in turn, activates ARF and leads to the expression of auxin response genes. ARF6 interacts with PIF4 and BZR1 (also known as the BZR1-ARF6-PIF4 (BAP) module) to coordinate plant growth regulation [52,54]. In this study, PP2C and SnRK2 genes were up-regulated at the transcriptional level. The expression of the ion transport genes AKT2/3 (Zm00001eb288580) and KAT2 (Zm00001eb120190) for sodium and potassium ion transport was down-regulated [55]. AKT2 and KAT2 are inward K^+^ channels. Up-regulation of AKT2/3 genes occurs in brine-exposed plants [56]. Studies have shown that PP2CA can inhibit the inward K^+^ channel activity of AKT2, thereby facilitating the inward flow of ions [57]. Meanwhile, AKT2 can regulate the sucrose/H^+^ symporter in Arabidopsis, which affects the entry of sucrose into the phloem [58]. RT-qPCR results showed down-regulation of the AKT2/3 gene after externally applied BS hormone, which verified the expression of this gene (Figure 10). It is speculated that BR and SL can jointly regulate the ABA signal pathway, regulate stomatal closure, and improve salt tolerance of maize. The DELLA protein controls the stability of the PIF protein. In this study, the expression of the DELLA protein gene Zm00001eb339390 was down-regulated, which positively regulated PIF4 activity and promoted the growth of maize. In this study, the expression of Zm00001eb153330 (probable WRKY transcription factor 65) was down-regulated. In Arabidopsis, the overexpression of NtWRKY65 enhanced the salt tolerance of transgenic plants, and its cis-acting elements responded to IAA, ABA, and SA [59]. Under cold stress, it is indicated that WRKY41, functioning as a substrate of MAX2, is negatively regulated in the freezing tolerance of Arabidopsis through MAX2-mediated ubiquitination and degradation. Furthermore, cold stress intensifies the interaction between WRKY41 and MYC genes, facilitates the synthesis of anthocyanins, and enhances the freezing tolerance of plants [60]. It is hypothesized that exogenous SL negatively regulating the down-regulated expression of WRKY65, in combination with the IAA and ABA signaling pathways, might reduce the accumulation of H_2_O_2_, facilitate the accumulation of anthocyanins, and enhance the activities of CAT and SOD, thereby augmenting the tolerance of maize seedlings to salt stress.

It is notable that in this study, it was discovered that after exogenous BS application under salt treatment, glucose-6-phosphate isomerase 1 chloroplastic (Zm00001eb101090, phi2) and aconitase (Zm00001eb419110, aco2) in the TCA cycle were down-regulated. SLs play a key role in the transduction of sugar signaling molecules involved in the development of *Arabidopsis* seedlings [61]. GR24+NaCl treatment was also found to significantly reduce glucose levels in tomato [62]. The RT-qPCR results showed that the expression levels of phi and aco2 under BSN (1.65 nM BR + 1 µM SL + 180 mM NaCl) treatment were found to be significantly lower than those observed under BS treatment (Figure 10). Sucrose inhibits MAX2 activity [63], and aconitase catalyzes the conversion of citrate, an intermediate of the citrate cycle (TCA), which is involved in MAX2 signaling [64]. We contend that the interaction between BR and SL has a physiological-level correlation with the TCA cycle and jointly functions in sensing metabolic fluxes.

**Figure 11:**
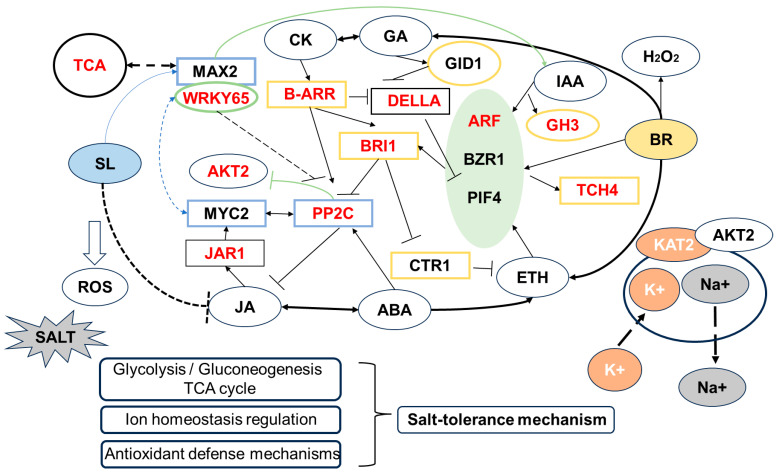
Interaction model of externally applied brassinosteroid (BR) and strigolactones (SLs) in response to salt stress in maize. The blue boxes denote exogenous SL response nodes, the yellow boxes signify exogenous BR response nodes, and the red fonts indicate exogenous BR-SL response nodes. Blue lines represent SLs regulation, green lines represent possible WRKY65 regulation. Dashed lines represent possible regulation.

In conclusion, the interaction between BR and SL takes place among multiple plant hormone signal nodes. Through the photosynthetic antenna protein pathway, it influences sugar metabolism and the citrate cycle, regulates ion transport, and mitigates salt stress. (Figure 11).

## 4. Materials and Methods

### 4.1. Experimental Materials

The salt-sensitive inbred line PH4CV and the salt-tolerant variety Zheng58 were provided by the corn research group of Agricultural College of Gansu Agricultural University were used as test materials. BR powder and SL powder were used in the experiment and were purchased from Beijing Solebao Technology Co., Ltd. (Beijing, China).

### 4.2. Experimental Design and Treatment

After screening the optimal hormone concentration according to the previous experiment, the two genotypes were treated with 8 treatments, which were distilled water (CK); 180 mM NaCl (N); 1.65 nM BR (BR); 0.1 mg/L BR + 180 mM NaCl (BRN); 1 µmol SL (SL); 1 µM SL + 180 mM NaCl (SLN); 1.65 nM BR + 1 µM SL (BS); 1.65 nM BR + 1 µM SL + 180 mM NaCl (BSN). The eight processes of PH4CV are named PCK, PN, PBR, PBRN, PSL, PSLN, PBS, and PBSN; the eight processes of Zheng58 are named ZCK, ZN, ZBR, ZBRN, ZSL, ZSLN, ZBS, and ZBSN in turn.

The maize germplasm genotype Zheng58 and PH4CV were provided by the Maize Breeding Research Group, College of Agronomy, Gansu Agricultural University. Following the addition of a 180 mM salt solution and vermiculite, corn seeds were planted in a pot with a diameter of 10 cm and were cultivated in the greenhouse. We poured 50 mL of the corresponding treatment solution every 2 days, and we took the penultimate leaf of corn seedlings at the V3 stage (about 14 days) for the determination of relevant indicators and subsequent analysis. There were three secondary physical repetitions for each treatment, with a total of 48 leaf samples.

### 4.3. Index Measurement

#### 4.3.1. Growth Index Measurement

After washing the vermiculite at the root of the seedlings, we blotted the surface moisture with filter paper. Seedling length (SDL) and root length (RL) of maize were measured with a straight ruler. Aboveground fresh weight (AW) and root fresh weight (RW) were measured by analytical balance.

#### 4.3.2. Measurement of Physiological Indexes

The activity of superoxide dismutase (SOD), peroxidase (POD), and catalase (CAT) were determined according to the method described by Zhang and Kirkham [65].

#### 4.3.3. Determination of Ion Content

Dried samples were digested using the H_2_SO_4_-H_2_O_2_ method in a Topwave microwave digestion analyzer (Analytik Jena, Germany). The digestion process was conducted in accordance with the operational procedures of the microwave digestion instrument. K and Na standard solutions were purchased from the National Center for Analysis and Testing of Nonferrous Metals and Electronic Materials. The contents of K and Na were determined by flame spectrophotometer (TAS-990, Beijing Puyang General Instrument Co., Ltd., Beijing, China) [66].

### 4.4. Transcriptomic Analysis

#### 4.4.1. Total RNA Extraction and Illumina Deep Sequencing

The total RNA of the 48 obtained leaf samples was extracted using TRIzol reagent. After extraction, Nanodrop2000 (Waltham, MA, USA) and Agient2100 (LabChip GX) (Santa Clara, CA, USA) instruments were used to detect the purity of the extracted total RNA, and RNA-Seq library was constructed for the sample RNA, conforming the conditions of the combined library. The library was constructed by Biomarker Technologies Co., Ltd. (Beijing, China).

#### 4.4.2. Quality Assessment of Sequencing Results

After the sequencing was completed, the quality control of the obtained raw reads was carried out, and high-quality clean data were obtained by filtering the original data. The obtained clean data were compared with the reference genome (Zm_B73_REFERENCE_GRAMENE_5.0) using the HISAT2 (2.1.0) software [67]. After the comparison was completed, the reads on the comparison were assembled and quantified by String Tie [68].

#### 4.4.3. Differential Expression Gene Analysis

Differential gene expression was analyzed using DESeq2 (4.3.2) [69]. The fold change ≥ 1.5 and *p*-value < 0.05 were used as the screening criteria to conduct differential gene analysis for all samples in this study. The differentially expressed genes were analyzed for GO (Gene Ontology), KOG (EuKaryotic Orthologous Groups), and KEGG (Kyoto Encyclopedia of Genes and Genomes) enrichment.

#### 4.4.4. Analysis of the Gene Co-Expression Network

Weighted gene co-expression network analysis (WGCNA) is a commonly used method for constructing gene co-expression networks. In this study, co-expression network analysis (FPKM = 1, fold threshold = 0.25) was performed for the gene expression of 48 sequenced samples under different treatments using the WGCNA tool in the Biomarker platform (www.biocloud.net) (accessed on 15 May 2024).

#### 4.4.5. Real-Time Fluorescence Quantitative PCR (RT-qPCR) Verification of DEGs

RNA was extracted from samples using the SteadyPure Plant RNA Extraction Kit. The RNA was then reverse transcribed using the Evo M-MLV RT Premix for qPCR. We performed real-time fluorescence PCR analysis using the SYBR Green Premix Pro Tag HS qPCR Kit (Rox Plus) and the QuantStudio 5 Real-Time PCR System (Thermo Scientific, Waltham, MA, USA) (Shanghai, China). All reagent kits were sourced from ACCURATE BIOTECHNOLOGY(HUNAN) CO., LTD, Changsha, China. Primer Premier 5.0 was used for primer design. The relative expression levels of the selected genes were calculated using the 2^−ΔΔCT^ method, using 3 biological replications per treatment [70].

### 4.5. Statistics

Excel 2019 was used to analyze and organize the data, and IBM SPSS 26.0 was used to organize and analyze the morphological and physiological data. RT-qPCR results were mapped using Origin 2021. Each experiment was repeated at least 3 times, and the values are shown as mean ± SE.

## 5. Conclusions

This study revealed that under salt stress, the spraying of 0.1 µmol/L exogenous SL and 1 mg/L exogenous BR and BS (1.65 nmol/L BR + 0.1 µM SL) solutions regulated ion balance, accelerated the efflux of Na^+^ from roots, improved the antioxidant capacity of maize, was conducive to maintaining the intracellular ionic homeostasis, and reduced oxidative damage caused by salt stress. Transcriptome analysis disclosed that the mitigation of salt stress by exogenous BR is intimately associated with the B-ARR family genes GID1, IAA hormone signaling, BRI1, and CTR1 and is implicated in the conversion of malic acid within the TCA cycle. Exogenous SL governs pathways such as Aux/IAA, MYC2, and ABA, as well as abiotic-stress-related transcription factors. There exists a synergistic effect between BR and SL. Through the interaction network of ARF-PIF4-BAR1, in conjunction with IAA, GA, ABA, and JA signaling pathways, it governs the elongation of the aboveground part of seedlings, regulates the activities of AKT2 and KAT2, and facilitates the influx of K^+^. It takes part in the TCA cycle by regulating aconitase and malic enzyme. WGCNA analysis results suggest that starch and sucrose metabolism, as well as fatty acid elongation, may be enriched pathways associated with maize seedling growth.

## Figures and Tables

**Table 1:** Effects of exogenous hormones on SOD, POD, and CAT enzyme activities of maize seedlings under salt stress.

Treatments	SOD Activity (U/g)	POD Activity (U/g)	CAT Activity (U/g)
ZCK	142.336 ± 18.597 e	29.778 ± 3.031 e	0.76 ± 0.062 e
ZN	254.594 ± 6.1 c	116 ± 14.967 cd	2.033 ± 0.21 d
ZBS	196.502 ± 10.565 d	124.445 ± 22.732 d	2.56 ± 0.333 d
ZBSN	273.489 ± 12.886 bc	233.334 ± 31.085 b	4.66 ± 0.086 bc
ZSL	192.889 ± 3.979 d	182.667 ± 31.284 c	4.94 ± 0.571 b
ZSLN	297.119 ± 8.247 b	206.223 ± 20.963 b	5.96 ± 0.353 a
ZBR	152.384 ± 12.616 e	135.556 ± 22.985 cd	2.18 ± 0.174 d
ZBRN	337.839 ± 15.161 a	276.889 ± 8.555 a	3.94 ± 0.405 c
PCK	131.707 ± 7.903 e	85.528 ± 3.195 d	1.227 ± 0.029 b
PN	290.244 ± 5.174 b	123.772 ± 18.504 bc	3.013 ± 0.171 b
PBS	185.366 ± 7.903 d	163.355 ± 22.709 c	1.693 ± 0.17 b
PBSN	307.317 ± 10.77 b	202.895 ± 4.881 b	3.334 ± 0.177 b
PSL	178.049 ± 7.903 d	192.74 ± 25.232 bc	1.687 ± 0.108 a
PSLN	292.683 ± 13.021 b	256.494 ± 11.281 a	2.187 ± 0.064 ab
PBR	241.463 ± 15.807 c	182.655 ± 8.654 bc	1.767 ± 0.153 b
PBRN	358.537 ± 7.9033 a	198.794 ± 3.289 b	2.32 ± 0.11 b

Note: The data are presented as the mean ± SD from independent experiments. Different lowercase letters indicate significant difference.

## Data Availability

The sequencing data of this study were stored in the SRA database of NCBI, and the accession number was PRJNA1109634.

## Supplementary Materials

The following supporting information can be downloaded at https://www.mdpi.com/article/10.3390/ijms251910505/s1.
